# Factorial invariance of the abridged version of the Explicit Discrimination Scale among adults living in southern Brazil

**DOI:** 10.1590/1980-549720240038

**Published:** 2024-07-15

**Authors:** Fabiula Renilda Bernardo, João Luiz Bastos, Michael Eduardo Reichenheim

**Affiliations:** I Universidade Federal de Santa Catarina, Graduate Program in Public Health – Florianópolis (SC), Brazil.; II Simon Fraser University, Faculty of Health Sciences – Burnaby, British Columbia, Canada.; III Universidade do Estado do Rio de Janeiro, Hésio Cordeiro Institute of Social Medicine – Rio de Janeiro (RJ), Brazil.

**Keywords:** Psychometrics, Intersectional framework, Perceived discrimination, Brazil

## Abstract

**Objective:**

The Explicit Discrimination Scale (EDS) was developed to assess experiences with discrimination in Brazilian epidemiologic surveys. Though previous analyses have demonstrated that the EDS has good configural, metric, and scalar properties, its invariance has not yet been investigated. In this study, we examined the factorial invariance of two abridged versions of the EDS, according to skin color/ethnicity, sex, socioeconomic status, and their intersections.

**Methods:**

Data from the EpiFloripa Adult Study were used, which include a representative sample of adults residing in a state capital of southern Brazil (n=1,187). Over half of the respondents were women, and around 90% identified as white; the mean age of the participants was 39 years. Two abridged versions of the EDS were analyzed, with seven and eight items, using Multigroup Confirmatory Analysis and the Alignment method.

**Results:**

The two versions of the scale may be used to provide estimates of discrimination that are comparable across skin color/ethnicity, sex, socioeconomic status, and their intersections. In the seven-item version of the scale, only one parameter lacked invariance (i.e., threshold of item i13 – called by names you do not like), specifically among black respondents with less than 12 years of formal education.

**Conclusion:**

The EDS may provide researchers with valid, reliable, and comparable estimates of discrimination between different segments of the population, including those at the intersections of skin color/ethnicity, sex, and socioeconomic status. However, future research is needed to determine whether the patterns we identified here are consistent in other population domains.

## INTRODUCTION

Until the 1990s, research on the health impacts of discrimination was scarce^1^. Since the 2000s, evidence on the relationships between discrimination, adverse health conditions, and related inequities has been accumulating^2^. The impact of discrimination includes damage to physical and mental health^3,4^, in addition to the adoption of risky behaviors and less frequent engagement with health promotion activities^5^. However, knowledge in the area has been limited by the lack of research conducted outside the United States of America^4^, limited analyses on discrimination among groups defined by sex, social class, race/ethnicity, and others^1,6^, and the emphasis on a single form of discrimination, especially race-based mistreatment^7^. The literature often disregards the fact that certain social groups lie at the intersection of multiple axes of oppression, which shape their experiences and worldviews^8^.

Intersectionality is an analytical framework that seeks to shed light on the intersections between different systems of oppression. In the field of public health, an intersectional approach highlights that social injustices do not operate in isolation, but are mutually produced, reinforced, and bear different meanings and intensities for varied population groups^6,9-11^. Moreover, social positions and identities (defined by social class, ethnicity, sex etc.) shape subjectivities, which underlie differences in how experiences of discrimination are interpreted and perceived^12^. Thus, analyzing the relationship between social injustices and health inequities from an intersectional perspective implies developing instruments that can measure discrimination across intersectional groups.

The development and evaluation of an instrument encompasses a range of activities such as the formulation of the underlying theoretical framework and the assessment of psychometric properties. When it comes to psychometric properties, the configural, metric, and scalar structures should be examined to provide evidence on the internal validity of the instrument. In addition to analyzing the various properties of these structures in the sample as a whole, it is worth investigating their invariance in different social groups^13,14^. It is the observation that these three structures are invariant between the groups in question that allows us to state that group differences are factual, and not due to the instruments’ internal problems^14-16^. In other words, invariance is a *sine qua non* condition for establishing that instruments are measuring the same construct, to the same extent, and at the same intensity among the various population groups. When invariance is not observed, the validity and comparability of scale-derived estimates may be called into question.^17^. Violation of measurement invariance is an important issue that may even help explain inconsistencies observed in the findings of previous studies. In the work by Bernardo, Bastos and Moretti-Pires^18^, for example, black women with high socioeconomic status had the lowest average score for perceived discrimination among all studied subgroups. In another study, Lee and Turney^19^ found that men had higher mean discrimination scores than women, including Hispanic women.

The Explicit Discrimination Scale (EDS)^20^ assesses perceived interpersonal discrimination. In particular, the EDS offers three main advantages when compared to other similar measures: first, it addresses a substantial number of potentially discriminatory situations; second, it allows respondents to attribute their experiences with differential treatment (i.e., mistreatment) to one or more factors; and, third, it assumes that mistreatment and its interpretation as a discriminatory event are related but distinct constructs. The scale originally consists of 18 items, in which respondents who indicate that they were mistreated are asked to answer three additional subitems:

1. The motivations attributed to the event, such as skin color/ethnicity, age, and social class, among others;
2. The degree of discomfort related to their perception; and
3. The interpretation of the event as discriminatory or not.

Since it was first developed, the EDS has been subjected to several psychometric evaluations^18,20-22^ Taken together, these studies suggest that the scale has robust configural, metric, and scalar properties. Items load strongly onto their respective dimensions, and are able to orderly position respondents along the latent trait *continuum*. Research also demonstrates that the factor structure of EDS is consistent across diverse populations, from undergraduate students to community adults, for both the self- and the interviewer-administered versions of the instrument. Recently, Bastos et al.^23^ showed that two abridged versions of the EDS, with either seven or eight items, had good configural, metric, and scalar properties. The main difference between them lies in the fact that the eight-item version has better coverage of the latent trait, comprehensively mapping the corresponding *continuum* of intensity. Nonetheless, there are no studies assessing the invariance of EDS in different social groups. Thus, to what extent the EDS is adequate to measure perceived discrimination and to establish valid comparisons between groups is unknown. In this study, we evaluate the factorial invariance of the two abridged versions of the EDS in different social groups, considering skin color/ethnicity, sex, socioeconomic status, and their intersections.

## METHODS

Data came from the second wave of the EpiFloripa Adult Study, which was conducted in 2012. The EpiFloripa Adult Study aimed to investigate social determinants of health in a representative sample of adults (20–59 years) from the urban area of Florianópolis, state of Santa Catarina, southern Brazil. The selection process at baseline (i.e., 2009) was carried out in two stages, and the final sample consisted of 1,720 participants. In 2012, all members of the baseline survey were invited to participate in a second study wave, of which 1,187 were effectively interviewed.

In both study waves, data collection was carried out through face-to-face interviews, conducted by previously-trained interviewers. To optimize and refine the process, a pilot study was carried out with approximately 100 adults in two census tracts that were not part of the final sample. Quality control was performed by reviewing and checking 15% of all interviews, selected at random. In-home interviews were conducted with the aid of Personal Digital Assistants. Detailed information on the data collection process can be found in a previous publication^24^.

### Study variables

The sample was analyzed according to sex, skin color/ethnicity, education, and the intersections of these variables. Sex was used to characterize participants as men or women. Skin color/ethnicity was collected according to the categories of the Brazilian Institute of Geography and Statistics: white, black, brown (i.e., the official term for Brazilian admixed populations), Asian, and Indigenous. Asian and Indigenous respondents were excluded from the analysis, while brown and black individuals were grouped into a single category, hereinafter referred to as “blacks.” Education was categorized into two strata of 0–11 and 12+ years of formal education, because they represent the division between high school and higher education in Brazil. The intersections between these variables were evaluated as: intersection of sex and education (Men/>11 years of education; Men/<12 years of formal education; Women/>11 years of formal education; Women/<12 years of formal education); intersection of skin color/ethnicity and education (Whites/>11 years of formal education; Whites/<12 years of formal education; Blacks/>11 years of formal education; Blacks/<12 years of formal education); and intersection of skin color/ethnicity and sex (Whites/Men; Whites/Women; Blacks/Men; Blacks/Women).

The analyses focus on the two abridged versions of the EDS, with seven and eight items^23^. The seven-item version contains the following: i2 (*treated with disrespect in public places*); i6 (*treated as unintelligent at school/university*); i7 (*treated as unintelligent at internship or work*); i9 (*unfairly evaluated at internship or work*); i13 (*called by names you do not like*); i14 (*left out by friends at school or university*); and i16 (*left out by people in the neighborhood*). The eight-item version also includes i15 (*left out by colleagues at internship or work*). All responses to the scale items were dichotomized into “yes,” when the respondent indicated the perception of differential treatment and attributed it to discrimination; in all other cases, items were categorized as “no.”

### Statistical analysis

Invariance of the EDS was analyzed using two complementary approaches: Multigroup Confirmatory Factor Analysis (MGCFA) and the Alignment method. Analyses based on the intersections of sex, skin color/ethnicity, and education were conducted only with the Alignment method. According to previous psychometric findings^20,21^, it was assumed that the EDS items reflect a one-dimensional configural structure.

MGCFA was conducted in three stages^16,25^. In the first stage, baseline models were estimated separately for each group of interest (i.e., men, women, whites, blacks etc.), and the fit indices RMSEA (Root Mean Square Error of Approximation), CFI (Bentler’s comparative fit index), and TLI (Tucker-Lewis index) were evaluated^16,25^. RMSEA values below 0.06 suggest a good fit; values above 0.10 indicate inadequate fit, and suggest that the model should be rejected. CFI and TLI above or equal to 0.95 indicate an acceptable fit^16^. In order to improve model fit, residual correlations between pairs of items were freely estimated, as suggested by the univariate Lagrange multiplier tests.

In the second stage, configural invariance was tested by freely estimating the factor loadings and thresholds in each group (configural model). The factor mean was set at zero and the factor scale was set at 1 in all groups. This was imposed to allow for model identification. The third stage consisted of comparing the configural model with the scalar model, in which factor loadings and thresholds were fixed between the groups. Considering that the EDS items were dichotomous, the Weighted Least Squares Mean and Variance (WLSMV) adjusted estimator was used^26^. It should be noted that the metric model is not identified when items are binary. This makes metric invariance testing impossible and implies that the comparison between the configural model and the fully restricted model (i.e., scalar model) is directly made^27^. The least and most restricted nested models were compared using robust chi-square statistics, adjusted for mean and variance^28^. A p-value lower than 0.05 suggests that the assumption of invariance between groups should be rejected. Variation in CFI values was also taken as an indication of invariance violation; when reductions above 0.002 were observed in the comparison of a less restrictive model with a more restrictive one, invariance violation was assumed^26,29^.

As indicated, invariance of the EDS was also analyzed according to the Alignment method^30^. Analyses were performed using the fixed option, which is recommended when comparing a few groups. There are two parameterizations available with the WLSMV estimator: Theta (unstandardized) and Delta (standardized). In order to achieve the best alignment (of factors to be compared) with a minimum of violations (i.e., the maximum number of fully-invariant items), Asparouhov and Muthén^30^ propose that the analyses be performed using both parameterizations, while opting for the one that offers the smallest number of non-invariance hits. Considering that the results were interchangeable, the authors decided to present only the data obtained with the Theta parameterization. The same fit indices used in the MGCFA were adopted to assess the models. Items with p>0.001 in the comparison of loadings and thresholds between groups were considered invariant^30^.

Database processing and sample description were performed using Stata, version 16.2. MGCFA and Alignment were run on Mplus, version 8.8. All statistical analyses were replicated for the seven- and eight-item versions of the EDS and took into account the complex sampling design and weights. The scripts for the analyses are available in the supplementary material.

### Ethical aspects

The EpiFloripa Adult Study was approved by its respective ethics committee, under Protocol No. 1772/11. Participation in the study was voluntary and all interviewees signed the Informed Consent Form.

## RESULTS

As can be seen in Table 1, the sample consisted of 56.9% women; about 89.7% of the interviewees identified as white; and 55.3% of the interviewees had up to 11 years of formal education. For the intersectional strata, whites with up to 11 years of formal education were the largest group (46.6%). There was a predominance of white women (51.7%) and women with up to 11 years of formal education (31.6%). Black men and women (5.1% in each group) were less frequent, especially those with a higher level of education (>11 years). The latter group accounted for only 1.8% of the total sample.

**Table 1:** Distribution of respondents according to sex, skin color/ethnicity, and education. Florianópolis (SC), 2012.

Characteristics	n*	%^†^	95%CI
Sex			
Men	504	43.1	40.5–45.8
Women	683	56.9	54.2–59.5
Skin color/Ethnicity			
White	1061	89.7	85.9–92.7
Blacks^‡^	122	10.3	7.3–14.1
Education (years of formal study)			
>11	525	44.7	37.3–52.3
<12	659	55.3	47.7–62.7
Intersection of sex and education (years of formal study)			
Men/>11	223	19.3	16.0–23.2
Men/<12	279	23.7	20.4–27.5
Women/>11	302	25.4	21.0–30.4
Women/<12	380	31.6	26.9–36.6
Intersection of skin color/ethnicity and education (years of formal study)			
Whites/>11	504	43.1	35.9–50.7
Whites/<12	554	46.6	39.7–53.6
Blacks/>11	21	1.8	1.1–2.8
Blacks/<12	101	8.5	5.9–12.1
Intersection of skin color/ethnicity and sex			
Whites/Men	447	38.1	35.5–40.7
Whites/Women	614	51.7	48.4–54.9
Blacks/Men	56	5.1	3.6–7.3
Blacks/Women	66	5.1	3.4–7.8

95%CI: 95% confidence interval.

* Absolute frequency;

^†^ Relative frequency, adjusted for sampling weights;

^‡^ Term used to represent the grouping of black and mixed-race individuals. The variables skin color/ethnicity and education presented, respectively, 4 and 3 ignored observations. The intersection of sex and education, skin color/ethnicity and education, and skin color/ethnicity and sex presented, respectively, 3, 7, and 4 missing observations.

MGCFA of the eight-item EDS indicated that the unifactorial model had a good fit to the data in all subgroups analyzed, with CFI≥0.95, TLI≥0.95, and RMSEA<0.06, except for participants with 12 or more years of formal education. When reviewing the values contained in Table 2, we observed that the latter model presented a borderline TLI, even after including the residual correlation between items i13 and i14. The χ2 difference test comparing the configural and scalar models among white and black participants indicated that the restriction to equal loadings and thresholds resulted in a nonsignificant increase in χ2. We also investigated the variation of the CFI and observed no reduction between more and less restrictive models. The sex-based comparison of the configural and scalar models was also not statistically significant, according to χ2; however, we identified a reduction of 0.003 in the CFI. Upon comparing participants with up to 11 and 12 or more years of formal education, the χ2 test was nonsignificant, with a CFI variation of 0.002. Modification indices did not show any violating item in any analyzed group.

**Table 2:** Multigroup Confirmatory Factor Analysis models for the Explicit Discrimination Scale, according to skin color/ethnicity, sex, and education. Florianópolis (SC), 2012.

	χ^2^	df	χ^2^diff	Δdf	RMSEA (90%CI)	CFit	CFI	TLI
Skin color/ethnicity								
Baseline models								
Whites	47.561*	20	-	-	0.036 (0.023-0.049)	0.958	0.964	0.950
Blacks	27.397	20	-	-	0.055 (0.000 - 0.102)	0.398	0.977	0.968
Invariance								
Configural	76.162*	40	-	-	0.039 (0.025-0.052)	0.909	0.965	0.950
Scalar (loadings and thresholds)	81.499*	46	8.861	6	0.036 (0.023-0.049)	0.966	0.965	0.958
Sex								
Baseline models								
Men	25.771	20	-	-	0.024 (0.000-0.048)	0.966	0.986	0.984
Women	31.981^†^	20	-	-	0.030 (0.005-0.048)	0.967	0.974	0.963
Invariance								
Configural	57.974^†^	40	-	-	0.028 (0.008-0.042)	0.996	0.982	0.974
Scalar (loadings and thresholds)	66.785^†^	46	10.556	6	0.028 (0.010-0.041)	0.998	0.979	0.974
Education (years of formal study)								
Baseline models								
>11^‡^	38.340^†^	19	-	-	0.044 (0.023-0.064)	0.661	0.959	0.940
<12	29.987	20	-	-	0.028 (0.000-0.047)	0.975	0.979	0.971
Invariance								
Configural	68.955^†^	39	-	-	0.036 (0.021-0.050)	0.953	0.969	0.955
Scalar (loadings and thresholds)	72.827*	45	6.334	6	0.032 (0.018-0.046)	0.988	0.971	0.964

90%CI: 90% confidence interval.

* p<0.001;

^†^ p<0.05;

^‡^ Model includes correlation between i14 and i13.

Note: χ^2^ (chi-square); df (degrees of freedom); χ^2^diff (χ^2^ difference); χ^2^df (difference between the degrees of freedom of the configural and scalar models); RMSEA (Root Mean Square Error of Approximation); CFit (test of close fit – probability of RMSEA=0.05); CFI (Comparative Fit Index) TLI (Tucker-Lewis Index).

As shown in Table 3, the results observed with the Alignment were consistent with those obtained with the MGCFA, suggesting invariance in the groups defined by sex, skin color/ethnicity, and education separately. The models showed a good fit, with CFI≥0.95, TLI≥0.95, and RMSEA<0.06, except in the group with 12 or more years of formal education. The latter model had a borderline TLI of 0.930. However, after including the residual correlation between items i13 and i14, the TLI value was 0.955. Item i9 could not be analyzed in the skin color/ethnicity groups because of estimation problems among blacks. This item presented loading and threshold values that were not admissible from an interpretative point of view due to the low frequency of positive responses and the small size of this group in the analysis.

**Table 3:** Factor loadings and thresholds of the Explicit Discrimination Scale. Florianópolis (SC), 2012.

Item	Non-standardized factor loadings				Thresholds			
	Skin color/ethnicity							
	Whites	Blacks	p-value	R²	Whites	Blacks	p-value	R²
i2	0.690	1.252	0.193	0.5	1.207	0.995	0.717	0.7
i6	1.026	1.694	0.387	0.5	2.042	2.417	0.704	0.7
i7	1.037	0.853	0.566	0.0	1.786	1.161	0.097	0.0
i9	0.825	8.056	0.808	0.2	1.723	10.058	0.821	0.3
i13	0.897	0.734	0.608	0.0	1.086	0.332	0.024	0.0
i14	1.067	1.026	0.899	0.9	1.493	1.531	0.915	1.0
i15	0.746	0.928	0.710	0.7	1.811	1.855	0.853	1.0
i16	0.671	0.665	0.965	1.0	2.064	1.662	0.325	0.0
	**Sex**							
	**Men**	**Women**	**p-value**	**R²**	**Men**	**Women**	**p-value**	**R²**
i2	1.096	0.670	0.103	0.4	1.465	1.331	0.569	1.0
i6	1.058	1.428	0.434	0.0	2.157	2.651	0.345	0.0
i7	1.389	0.981	0.264	0.5	2.344	1.794	0.151	0.7
i9	1.077	1.099	0.865	1.0	2.095	2.109	0.887	1.0
i13	0.820	1.085	0.470	0.0	1.033	1.393	0.260	0.0
i14	1.174	1.099	0.807	0.9	1.772	1.700	0.832	1.0
i15	0.777	1.011	0.492	0.0	1.787	2.320	0.135	0.0
i16	1.062	0.618	0.140	0.4	2.437	2.133	0.382	0.8
	Education							
	>11* years	**<12 years**	**p-value**	**R²**	**>11 years**	**<12 years**	**p-value**	**R²**
i2	0.68	0.785	0.614	0.8	1.228	1.043	0.271	0.0
i6	1.025	1.135	0.741	0.8	1.965	2.091	0.723	0.9
i7	1.344	0.778	0.063	0.0	2.003	1.446	0.047	0.0
i9	0.964	0.952	0.942	1.0	1.748	1.808	0.742	1.0
i13	0.577	0.721	0.526	0.6	0.836	0.851	0.830	1.0
i14	0.817	0.797	0.901	1.0	1.156	1.377	0.262	0.7
i15	0.961	0.666	0.280	0.0	1.867	1.780	0.715	0.0
i16	0.854	0.588	0.706	0.0	2.154	1.929	0.438	0.0

It was not possible to estimate loadings and thresholds for item i9 among blacks.

* Model includes correlation between i13 and i14.

According to data in Table 4, there is no invariance violation at the intersections of skin color/ethnicity and education, skin color/ethnicity and sex, sex and education for the eight-item EDS. At the intersection between “skin color/ethnicity and level of education,” the subgroup of blacks with more than 12 years of formal education was removed from the analyses. The substantially small subsample (i.e., 21 participants) for this group caused estimation problems, with loadings and thresholds that were not admissible from an interpretative point of view, and contingency tables without information.

**Table 4:** Factor loadings and thresholds of the Explicit Discrimination Scale*,* according to intersections. Florianópolis (SC), 2012.

Item	Sex and education									
	Non-standardized factor loadings					Thresholds				
	Men/>11	Men/<12	Women/<11^†^	Women/<12	R²	Men/>11	Men/<12	Women/>11	Women/<12	R²
i2	1.251	1.154	0.544	1.003	0.5	1.826	1.099	1.088	1.116	0.0
i6	0.836	1.200	1.465	1.739	0.0	1.933	2.076	2.173	2.219	0.7
i7	1.399	1.330	1.389	0.930	0.6	2.323	2.038	1.807	1.291	0.1
i9	0.870	1.363	1.313	1.194	0.1	1.890	2.140	1.830	1.723	0.7
i13	0.759	0.759	0.776	1.205	0.0	0.956	0.876	0.923	0.939	0.8
i14	1.278	1.025	0.893	1.036	0.7	1.742	1.529	1,071	1.345	0.5
i15	0.951	0.750	1.335	1.028	0.0	1.830	1.689	2.150	1.959	0.0
i16	1.048	1.139	1.048	0.395	0.4	2.532	2.223	2.167	1.865	0.1
	**Skin color/ethnicity and education**									
	**Whites/>11** *	**Whites/<12**	**Blacks/<12**	**R²**		**Whites/>11**	**Whites/<12**	**Blacks/<12**	**R²**	
i2	0.704	0.800	1.569	0.5		1.247	1.084	1.137	0.8	
i6	1.020	1.118	1.512	0.8		1.951	1.989	2.370	0.7	
i7	1.281	1.001	0.561	0.0		1.926	1.621	0.933	0.0	
i9	0.900	0.830	3.236	0.4		1.681	1.660	4.530	0.3	
i13	0.583	0.933	0.594	0.0		0.856	1.013	0.297	0.0	
i14	0.840	0.922	0.936	1.0		1.147	1.409	1.465	0.8	
i15	0.961	0.741	0.670	0.0		1.848	1.815	1.613	0.0	
i16	0.865	0.664	0.643	0.3		2.215	1.965	1.868	0.0	
	**Skin color/ethnicity and sex**									
	**White men**	**White women**	**Black men**	**Black women**	**R²**	**White men**	**White women**	**Black men**	**Black women**	**R²**
i2	1.029	0.640	1.243	1.430	0.6	1.460	1.210	1.160	1.095	0.6
i6	0.923	1.299	1.535	1.943	0.6	2.063	2.233	2.509	2.742	0.7
i7	1.310	1.044	1.287	0.769	0.5	2.370	1.657	1.618	1.158	0.0
i9	0.801	1.034	10.903	7.227	0.3	1.904	1.797	13.006	11.136	0.3
i13	0.875	1.084	0.508	0.761	0.0	1.178	1.191	0.155	0.617	0.0
i14	1.153	1.131	1.133	0.988	0.9	1.745	1.477	1.854	1.476	0.7
i15	0.712	0.993	0.935	1.150	0.6	1.766	2.064	1.643	2.708	0.3
i16	1.262	0.507	0.416	1.242	0.0	2.707	1.973	1.710	2.027	0.0

It was not possible to estimate loadings and thresholds for item i9 among blacks with up to 11 years of formal education.

* Model includes correlation between i13 and i14;

^†^ Model includes correlation between i13 and i14.

The analyses previously described were replicated for the seven-item EDS. In the MGCFA, the baseline models of each group also showed an acceptable fit, with CFI≥0.95, TLI≥0.95, and RMSEA<0.06. Once again, item i9 presented estimation problems in the configural model for blacks. The test to compare the configural and scalar models (χ2diff=11.666; Δdf=5; p=0.04) between white and black participants showed a significant increase in the chi-square test, and we also verified a reduction in the CFI of 0.004 between the more and less restricted models. The χ2 test comparing the configural and scalar models was not statistically significant, but we observed reductions in the CFI of 0.004 and 0.005 for sex- and education-based comparisons. Modification indices were examined, and no items with invariance violations were identified (see Table 1, supplementary material).

The Alignment method was also used to evaluate the seven-item EDS. The fit of the models was acceptable, with CFI≥0.95, TLI≥0.95, and RMSEA<0.06. Loadings and thresholds were not significantly different between whites and blacks, indicating invariance between the skin color/ethnicity groups. Nevertheless, item i9 presented the same problem as that observed for the eight-item scale, and was not subject to consideration for the comparison in question. All seven items were considered invariant according to sex and education (see Table 2, supplementary material). In addition, the seven items were invariant among all intersectional subgroups (see Table 3, supplementary material), with the exception of i13 (*called by names you do not like*), which was considered to be non-invariant for the intersection “skin color/ethnicity and education” among black participants with less than 12 years of formal education. Estimation problems observed for i9 (i.e., *unfairly evaluated at internship or work*) remained in the seven-item version of the scale.

**Figure 1:**
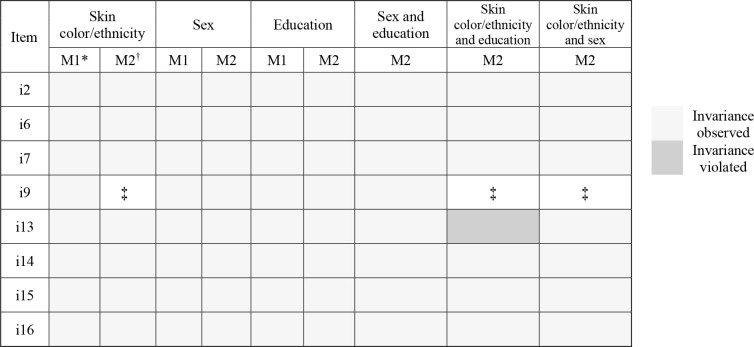
Summary of the invariance tests of the Explicit Discrimination Scale.

In Figure 1, we provide a summary of the study findings, showing that the two abridged versions of the EDS were invariant according to both the MGCFA and the Alignment method.

*Method 1 (M1): Multigroup Confirmatory Factor Analysis; ^†^Method 2 (M2): Alignment method; ^‡^Estimates for i9 could not be provided in the analysis of the skin color/ethnicity group, including its intersection with education and sex.

## DISCUSSION

Discriminatory experiences and their health impacts on various social groups have been captured by some scales available in the literature^20^. The EDS, in particular, has been used in Brazilian studies^31,32^, and previous psychometric evaluations lend credence to its validity and reliability^20-22^. Still, the fundamental assumption of measurement invariance across social and intersectional groups has remained, until now, without any critical appreciation. In this study, we identified, for two abridged versions of the EDS, configural and scalar invariance between groups defined by sex, skin color/ethnicity, and education, indicating that the instrument provides comparable discrimination estimates across them. Our findings also suggest that the two abridged versions of the EDS are invariant across intersectional groups, as defined by sex, skin color/ethnicity, and education. We found only one non-invariant parameter (threshold; item i13, called by names you do not like) in the seven-item EDS, indicating that the eight-item version is more suitable for use. This is also justified by the better coverage of the latent trait of the eight-item EDS, as evidenced in a previous study^23^.

The results were consistent when two different methodological approaches were employed (i.e., MGCFA and Alignment). The few exceptions refer to i9 for the comparisons by skin color/ethnicity; the intersections between “skin color/ethnicity and sex” and “skin color/ethnicity and education.” The low percentage of positive responses to some items had already been observed in a previous publication^20^. This issue proved to be even more problematic in MGCFA, especially in a sparse stratum such as that of blacks. In addition to the low endorsement of i9, in order to proceed with invariance analyses, the residual correlation between i14 and i13 was estimated for the groups with more than 11 years of education; whites with more than 11 years of education and women with more than 11 years of education. We also identified a residual correlation between items i9 and i7 for whites, the latter being observed only in the seven-item EDS. Taking this into consideration, our results demonstrate that the two methods used in the analyses are mutually supportive. However, our analyses should be replicated in a future study, preferably with a larger sample size to tackle the issue of low item endorsement.

Taken together, our findings support use of the two abridged versions of the EDS to investigate discrimination in different groups, defined by sex, skin color/ethnicity, and education. Nevertheless, two issues should be considered and addressed in future studies: first, the relatively small proportion of blacks in the sample; second, the data analyzed here come from adults living in the city of Florianópolis, and there is, therefore, a need to conduct additional research to determine whether the identified patterns emerge and are confirmed in other population domains. Brazil has a wide cultural and sociodemographic diversity throughout its extensive territorial area. Thus, perceptions of and experiences with discrimination may be markedly different in other regions, with implications for how they should be measured using psychometric scales.

Despite the aforementioned limitations and the need for additional tests, the strategies employed here allowed us to conclude that the abridged versions of the scale, as proposed by Bastos et al.^23^, are invariant between the studied groups. Therefore, the present study contributes to the further development of a discrimination scale for use in Brazil with high levels of validity, reliability, and comparability.
