# Supplementary material for: Factorial invariance of the abridged version of the Explicit Discrimination Scale among adults living in southern Brazil
Source: Rev Bras Epidemiol. 2024 Jul 15;27:e240038. doi: 10.1590/1980-549720240038 (PMC11251642; doi:10.1590/1980-549720240038)
Supplement: Supplementary file 1 [file 1980-5497-rbepid-27-e240038-sup1.pdf]

MATERIAL SUPLEMENTAR

Tabela 1. Índices de ajuste da análise fatorial confirmatória multigrupo para a Escala de Discriminação Explícita (7 itens), de acordo com cor/raça, sexo e escolaridade. Estudo EpiFloripa, Florianópolis, 2012.

	χ2	df	χ2diff	Δdf	RMSEA (IC 90% [a])	CFit	CFI	TLI
Cor/raça								
Modelos basilares								
Brancos [b]	22,860*	13	-	-	0,027 (0,005 - 0,044)	0,987	0,986	0,978
Negros	19,992	14	-	-	0,059 (0,000 - 0,113)	0,357	0,983	0,974
Invariância								
Configural	44,210*	27	-	-	0,033 (0,013 - 0,050)	0,953	0,983	0,974
Escalar (cargas e limiares)	54,109**	32	11,666*	5	0,034 (0,017 - 0,050)	0,955	0,979	0,972
Sexo								
Modelos basilares								
Homens	18,586	14	-	-	0,025 (0,000 - 0,053)	0,922	0,990	0,986
Mulheres	26,355*	14	-	-	0,036 (0,013 - 0,057)	0,855	0,970	0,955
Invariância								
Configural	45,023*	28	-	-	0,032 (0,012 - 0,049)	0,963	0,981	0,972
Escalar (cargas e limiares)	54,015*	33	10,162	5	0,033 (0,016 - 0,048)	0,970	0,977	0,970
Escolaridade								
Modelos basilares								
>11 anos de estudo [c]	23,792*	13	-	-	0,040 (0,011 - 0,065)	0,723	0,973	0,956
<12 anos de estudo	18,925	14	-	-	0,023 (0,000 - 0,047)	0,970	0,989	0,984
Invariância								
Configural	42,452*	27	-	-	0,031 (0,010 - 0,048)	0,966	0,982	0,972
Escalar (cargas e limiares)	51,635*	32	9,883	5	0,032 (0,014 - 0,048)	0,971	0,977	0,970

[a] IC 90% = Intervalo de confiança de 90%. [b] Modelo inclui correlação entre o i9 e i7. [c] Modelo inclui correlação entre o i14 e i13. Nota: χ2 (*chi-square*); df (*degrees of freedom*); χ2diff (χ2 *difference*); Δdf (diferença entre *degrees of freedom* do modelo configural e escalar); RMSEA (*Root Mean Square Error of Approximation*); CFit (*test of close fit* – probabilidade RMSEA ≤ 0,05); CFI (*Comparative Fit Index*) TLI (*Tucker-Lewis Index*). ** $p < 0,001$ * $p < 0,05$.

Tabela 2. Cargas fatoriais não padronizadas e limiares da Escala de Discriminação Explícita (7 itens), de acordo com cor/raça, sexo e escolaridade. Estudo EpiFloripa, Florianópolis, 2012.

Item	Cargas fatoriais não padronizadas				Limiares			
	Cor/raça							
	Brancos	Negros	p-valor	R^2	Brancos	Negros	p-valor	R^2
i2	0,704	1,231	0,366	0,556	1,215	1,171	0,788	0,994
i6	1,034	1,620	0,508	0,617	2,051	2,653	0,587	0,541
i7	1,036	0,662	0,230	0,000	1,785	1,168	0,125	0,000
i9	0,764	8,274	0,853	0,262	1,672	12,115	0,859	0,168
i13	0,941	0,678	0,457	0,000	1,110	0,415	0,038	0,000
i14	1,083	0,991	0,829	0,766	1,505	1,676	0,672	0,848
i16	0,599	0,637	0,734	0,966	1,999	1,753	0,533	0,000
	Sexo							
	Homens	Mulheres	p-valor	R^2	Homens	Mulheres	p-valor	R^2
i2	1,151	0,697	0,146	0,462	1,506	1,334	0,519	0,943
i6	1,094	1,473	0,493	0,000	2,197	2,658	0,404	0,000
i7	1,367	0,988	0,388	0,587	2,319	1,774	0,209	0,696
i9	0,982	1,077	0,798	0,000	1,997	2,052	0,729	0.984
i13	0,847	1,157	0,490	0,000	1,047	1,421	0,275	0,000
i14	1,181	1,140	0,680	0,973	1,778	1,707	0,805	0,980
i16	1,012	0,533	0,169	0,397	2,376	2,034	0,328	0,746
	Escolaridade							
	>11[a] anos de estudo	<12 anos de estudo	p-valor	R^2	>11 anos de estudo	<12 anos de estudo	p-valor	R^2
i2	0,683	0,793	0,653	0,653	1,230	1,029	0,291	0,000
i6	1,062	0,793	0,865	0,978	2,001	2,079	0,807	0,966
i7	1,644	0,654	0,017	0,000	2,302	1,356	0,017	0,000
i9	0,869	0,871	0,988	1,000	1,668	1,740	0,734	0,961
i13	0,607	0,689	0,727	0,864	0,847	0,817	0,808	0,995
i14	0,844	0,730	0,658	0,000	1,172	1,325	0,432	0,891
i16	0,640	0,564	0,743	0,000	1,945	1,911	0,861	0,911

Para o i9, não foi possível estimar adequadamente cargas não padronizadas e limiares para o grupo negros, ver seção de resultados. [a] Modelo inclui correlação entre i13 e i14.

Tabela 3. Cargas fatoriais não padronizadas e limiares da Escala de Discriminação Explícita (7 de itens), de acordo com a combinação das variáveis cor/raça, sexo e escolaridade. Estudo EpiFloripa, Florianópolis, 2012.

Item	Cargas fatoriais não padronizadas					Limiares				
	**Intersecção de gênero e escolaridade**									
	Homens/>11 anos de estudo	Homens/ <12 anos de estudo	Mulheres/ >11 anos de estudo	Mulheres/ <12 anos de estudo	R^2	Homens/>11 anos de estudo	Homens/ <12 anos de estudo	Mulheres/ >11 anos de estudo	Mulheres/ <12 anos de estudo	R^2
i2	1,312	1,278	0,455	1,285	0,434	1,881	1,017	1,045	1,147	0,000
i6	0,901	1,284	1,332	2,185	0,000	1,997	1,977	2,015	2,332	0,574
i7	1,535	1,264	1,312	0,934	0,702	2,477	1,805	1,713	1,236	0,020
i9	0,623	1,466	1,160	1,309	0,000	1,680	2,033	1,683	1,678	0,706
i13	0,810	0,830	1,340	1,400	0,000	0,981	0,815	1,168	0,930	0,000
i14	1,370	1,105	1,614	1,138	0,413	1,822	1,447	1,437	1,315	0,302
i16	0,934	1,223	0,648	0,516	0,507	2,393	2,133	1,829	1,877	0,389
	**Intersecção de cor/raça e escolaridade**									
	Cargas fatoriais não padronizadas					Limiares				
	Brancos/>11 anos de estudo	Brancos/ <12 anos de estudo	Negros/<12 anos de estudo		R^2	Brancos/>11 anos de estudo	Brancos/ <12 anos de estudo	Negros/ <12 anos de estudo		R^2
i2	0,656	0,779	1,409		0,624	1,219	1,092	1,107		0,843
i6	0,967	1,019	1,317		0,848	1,899	1,979	2,309		0,777
i7	1,275	0,793	0,422		0,000	1,920	1,524	0,887		0,000
i9	0,733	0,711	2,985		0,407	1,549	1,617	4,673		0,281
i13	0,893	0,896	0,509		0,000	0,991	1,016	0,278*		0,000
i14	1,293	0,804	0,823		0,000	1,436	1,374	1,442		0,959
i16	0,589	0,616	0,578		0,985	1,943	1,966	1,869		0,817
	**Intersecção de cor/raça e gênero**									
	Cargas fatoriais não padronizadas					Limiares				
	Brancos/Homens	Brancos/Mulheres	Negros/Homens	Negros/Mulheres	R^2	Brancos/Homens	Brancos/Mulheres	Negros/Homens	Negros/Mulheres	R^2
i2	1,080	0,670	1,325	2,083	0,372	1,498	1,244	1,025	1,420	0,345
i6	0,962	1,327	1,651	2,338	0,391	2,104	2,274	2,411	2,971	0,621
i7	1,338	1,078	1,083	0,825	0,593	2,402	1,703	1,279	1,182	0,000
i9	0,679	1,029	8,385	5,270	0,293	1,796	1,809	9,056	7,528	0,327
i13	0,921	1,186	0,519	0,854	0,000	1,205	1,282	0,089	0,660	0,000
i14	1,159	1,189	1,180	1,169	0,998	1,752	1,543	1,716	1,574	0,922
i16	1,168	0,415	0,441	1,175	0,000	2,585	1,919	1,670	1,882	0,000

*$p<0,001$. Nota. Para o i9, não foi possível estimar adequadamente cargas não padronizadas e limiares para os grupos negros com até 11 anos de estudo formal; homens negros; mulheres negras

Script das análises de invariância, conforme sintaxe do Mplus.
O exemplo abaixo refere-se aos testes de invariância, conforme escolaridade dos respondentes, em contexto de Análise Fatorial Multigrupo. As demais comparações realizadas no artigo seguiram rotina semelhante.

Estimação do modelo basilar para respondentes com alta escolaridade

```
TITLE:
   Analise da invariancia da EDE (8 itens)
   AFC para grupo 12 anos e mais

DATA:
   FILE IS Banco_Epifloripa.dat;

VARIABLE:
   NAMES ARE
   ident      !Identificacao
   setor      !Unidades de amostragem
   peso       !Pesos amostrais
   ed2g1 ed2g2 ed2g3 ed2g4 ed2g5 ed2g6
   ed2g7 ed2g8 ed2g9 ed2g10 ed2g11 ed2g12
   ed2g13 ed2g14 ed2g15 ed2g16 ed2g17 ed2g18
   cor2 sexo2 escolar gencorescolar1 genescolar1;

   SUBPOPULATION ARE escolar EQ 0; !0==12 e mais/Alta

   USEVARIABLES ARE
   ed2g2 ed2g6 ed2g7 ed2g9
   ed2g13 ed2g14 ed2g15 ed2g16;

   CATEGORICAL ARE
   ed2g2 ed2g6 ed2g7 ed2g9
   ed2g13 ed2g14 ed2g15 ed2g16;

   MISSING = .;

   CLUSTER = setor;
   WEIGHT = peso;

ANALYSIS:
   TYPE = COMPLEX;
   ESTIMATOR = WLSMV;

MODEL:
   F1 BY ed2g2 ed2g6 ed2g7 ed2g9
   ed2g13 ed2g14 ed2g15 ed2g16;

ed2g14 with ed2g13;

OUTPUT:
   SAMPSTAT ;
   MODINDICES(all);
   STAND;
   RESIDUAL;
```

```
   TECH2;
```

Estimação do modelo basilar para respondentes com baixa escolaridade

```
TITLE:
   Analise da invariancia da EDE (8 itens)
   AFC para grupo ate 11 anos

DATA:
   FILE IS Banco_Epifloripa.dat;

VARIABLE:
   NAMES ARE
   ident       !Identificacao
   setor       !Unidades de amostragem
   peso        !Pesos amostrais
   ed2g1 ed2g2 ed2g3 ed2g4 ed2g5 ed2g6
   ed2g7 ed2g8 ed2g9 ed2g10 ed2g11 ed2g12
   ed2g13 ed2g14 ed2g15 ed2g16 ed2g17 ed2g18
   cor2 sexo2 escolar gencorescolar1 genescolar1;

   SUBPOPULATION ARE escolar EQ 1; !1==Ate 11 anos/Baixa

   USEVARIABLES ARE
   ed2g2 ed2g6 ed2g7 ed2g9
   ed2g13 ed2g14 ed2g15 ed2g16;

   CATEGORICAL ARE
   ed2g2 ed2g6 ed2g7 ed2g9
   ed2g13 ed2g14 ed2g15 ed2g16;

   MISSING = .;

   CLUSTER = setor;
   WEIGHT = peso;

ANALYSIS:
   TYPE = COMPLEX;
   ESTIMATOR = WLSMV;

MODEL:
   F1 BY ed2g2 ed2g6 ed2g7 ed2g9
   ed2g13 ed2g14 ed2g15 ed2g16;

OUTPUT:
   SAMPSTAT;
   MODINDICES(all);
   STAND;
   RESIDUAL;
   TECH2;
```

Análise de invariância configural

```
TITLE:
   Analise da invariancia da EDE (8 itens) entre
   12 anos e mais e ate 11 anos de escolaridade
   Invariancia configural

DATA:
   FILE IS Banco_Epifloripa.dat;

VARIABLE:
   NAMES ARE
   ident       !Identificacao
   setor       !Unidades de amostragem
   peso        !Pesos amostrais
   ed2g1 ed2g2 ed2g3 ed2g4 ed2g5 ed2g6
   ed2g7 ed2g8 ed2g9 ed2g10 ed2g11 ed2g12
   ed2g13 ed2g14 ed2g15 ed2g16 ed2g17 ed2g18
   cor2 sexo2 escolar gencorescolar1 genescolar1;

   USEVARIABLES ARE
   ed2g2 ed2g6 ed2g7 ed2g9
   ed2g13 ed2g14 ed2g15 ed2g16;

   CATEGORICAL ARE
   ed2g2 ed2g6 ed2g7 ed2g9
   ed2g13 ed2g14 ed2g15 ed2g16;

   MISSING = .;

   GROUPING IS escolar (0=Alta 1=Baixa); !Alta=12 e mais Baixa
=ate 11 anos

   CLUSTER = setor ;
   WEIGHT = peso ;

ANALYSIS:
  TYPE = COMPLEX;
  ESTIMATOR = WLSMV;

MODEL:
  F1 BY ed2g2 ed2g6 ed2g7 ed2g9
        ed2g13 ed2g14 ed2g15 ed2g16;

  [F1@0];        !Media do fator fixada em 0 em todos os grupos
  {ed2g2-ed2g16@1}; !Fator da escala fixada em 1 em todos os
grupos

  ed2g14 with ed2g13;

MODEL Baixa:
    F1 BY ed2g2@1 ed2g6 ed2g7 ed2g9
         ed2g13 ed2g14 ed2g15 ed2g16;
```

```
    [ed2g2$1-ed2g16$1];

    ed2g14 with ed2g13@0;


MODEL Alta:

    ed2g14 with ed2g13;

OUTPUT:
    SAMPSTAT;
    MODINDICES(all);
    STAND ;
    RESIDUAL ;
    TECH2 ;

SAVEDATA:
DIFFTEST = configescolar.dat;
```

Invariância escalar

```
TITLE:
   Analise da invariancia da EDE (8 itens) entre
   12 anos e mais e ate 11 anos de escolaridade
   Invariancia escalar
DATA:
   FILE IS Banco_Epifloripa.dat;

VARIABLE:
   NAMES ARE
   ident      !Identificacao
   setor      !Unidades de amostragem
   peso       !Pesos amostrais
   ed2g1 ed2g2 ed2g3 ed2g4 ed2g5 ed2g6
   ed2g7 ed2g8 ed2g9 ed2g10 ed2g11 ed2g12
   ed2g13 ed2g14 ed2g15 ed2g16 ed2g17 ed2g18
   cor2 sexo2 escolar gencorescolar1 genescolar1;

   USEVARIABLES ARE
   ed2g2 ed2g6 ed2g7 ed2g9
   ed2g13 ed2g14 ed2g15 ed2g16;

   CATEGORICAL ARE
   ed2g2 ed2g6 ed2g7 ed2g9
   ed2g13 ed2g14 ed2g15 ed2g16;

   MISSING = .;

   GROUPING IS escolar(0 = Alta 1 = Baixa) ; !Alta=12 e mais
Baixa= Ate 11 anos

   CLUSTER = setor;
   WEIGHT = peso;

```

```
ANALYSIS:
   TYPE = COMPLEX;
   ESTIMATOR = WLSMV;
   DIFFTEST = configescolar.dat;

MODEL:
  F1 BY ed2g2 ed2g6 ed2g7 ed2g9
       ed2g13 ed2g14 ed2g15 ed2g16;

  ![F1@0]; !Media do fator fixada em 0 em todos os grupos
  !{ed2g2-ed2g16@1}; !fator da escala fixada em 1 em todos os
grupos

  ed2g14 with ed2g13;

 MODEL Baixa:
    ed2g14 with ed2g13@0;

 MODEL Alta:
    ed2g14 with ed2g13;

OUTPUT:
    SAMPSTAT;
    MODINDICES(all);
    STAND;
    RESIDUAL;
    TECH2;
```

O exemplo abaixo refere-se aos testes de invariância, conforme escolaridade dos respondentes, em contexto de *Alignment*. As demais comparações realizadas no artigo seguiram rotina semelhante.

```
TITLE:
   Modelo(alignment_fixed) para a EDE (8 itens)
     ***Metodo Alignment ***

DATA:
   FILE IS Banco_Epifloripa.dat;

VARIABLE:
   NAMES ARE
   ident      !Identificacao
   setor      !Unidades de amostragem
   peso       !Pesos amostrais
   ed2g1 ed2g2 ed2g3 ed2g4 ed2g5 ed2g6
   ed2g7 ed2g8 ed2g9 ed2g10 ed2g11 ed2g12
   ed2g13 ed2g14 ed2g15 ed2g16 ed2g17 ed2g18
   cor2 sexo2 escolar gencorescolar1 genescolar1;

   USEVARIABLES ARE
   ed2g2 ed2g6 ed2g7 ed2g9
   ed2g13 ed2g14 ed2g15 ed2g16;
```

```
   CATEGORICAL ARE
   ed2g2 ed2g6 ed2g7 ed2g9
   ed2g13 ed2g14 ed2g15 ed2g16;

   KNOWNCLASS  IS  g(escolar=0  escolar=1);  !0==12  anos  e
mais/Alta 1==Ate 11 anos/Baixa
   CLASSES ARE g(2);

   MISSING = .;

   CLUSTER = setor;
   WEIGHT = peso;

ANALYSIS:
  TYPE = COMPLEX MIXTURE;
  ESTIMATOR IS MLR;
  ALGORITHM = INTEGRATION;
  !MODEL = CONFIGURAL METRIC SCALAR;
  ALIGNMENT = FIXED; !FREE;

MODEL:
  %OVERALL%
  f1 BY ed2g2 ed2g6 ed2g7 ed2g9
   ed2g13 ed2g14 ed2g15 ed2g16;

OUTPUT:
   !stand(STDYX);
   TECH1;
   TECH8;
   ALIGN;

PLOT:
  TYPE IS PLOT2;
```
