# Supplementary material for: Factorial invariance of the abridged version of the Explicit Discrimination Scale among adults living in southern Brazil
Source: Rev Bras Epidemiol. 2024 Jul 15;27:e240038. doi: 10.1590/1980-549720240038 (PMC11251642; doi:10.1590/1980-549720240038)
Supplement: Translation

# Translation: Invariância fatorial da versão abreviada da Escala de Discriminação Explícita em adultos do sul do Brasil

**DOI:** 10.1590/1980-549720240038.2

**Authors:** Fabiula Renilda Bernardo, João Luiz Bastos, Michael Eduardo Reichenheim

**Affiliations:** I Universidade Federal de Santa Catarina, Programa de Pós-Graduação em Saúde Coletiva – Florianópolis (SC), Brasil.; II Simon Fraser University, Faculty of Health Sciences – Burnaby, British Columbia, Canadá.; III Universidade do Estado do Rio de Janeiro, Instituto de Medicina Social Hésio Cordeiro – Rio de Janeiro (RJ), Brasil.

**Keywords:** Psicometria, Enquadramento interseccional, Discriminação percebida, Brasil

## Abstract

**Objective:**

A Escala de Discriminação Explícita (EDE) foi desenvolvida para avaliar experiências com discriminação em inquéritos epidemiológicos no Brasil. Embora análises prévias tenham revelado boas propriedades configurais, métricas e escalares do instrumento, nenhum estudo examinou sua invariância. Este trabalho objetivou examinar a invariância fatorial de duas versões abreviadas da EDE, considerando cor/raça, sexo, posição socioeconômica e suas intersecções.

**Métodos:**

Utilizaram-se dados do Estudo EpiFloripa Adulto, que encerra uma amostra representativa de residentes de uma capital do sul do Brasil (n=1.187). Cerca de 57% da amostra foi constituída por mulheres e 90% dos entrevistados se declararam brancos; a média de idade dos participantes foi de 39 anos. Duas versões abreviadas da EDE, com sete e oito itens, foram examinadas por meio de Análises Fatoriais Confirmatórias Multigrupo e o método **Alignment**.

**Resultados:**

As duas versões da escala produziram estimativas comparáveis de discriminação entre grupos definidos por cor/raça, sexo, posição socioeconômica e suas intersecções. Na versão reduzida de sete itens, apenas um parâmetro apresentou violação de invariância (limiar do item i13; i.e., chamado por nomes que não gosta), especificamente no grupo de respondentes negros com menos de 12 anos de escolaridade.

**Conclusão:**

Os resultados mostraram que a EDE é capaz de produzir estimativas de discriminação válidas, confiáveis e comparáveis entre diversos segmentos da população, incluindo aqueles situados na intersecção de cor/raça, sexo e posição socioeconômica. Contudo, pesquisas futuras são necessárias para verificar se os padrões identificados aqui podem ser confirmados em outros domínios populacionais.

## INTRODUÇÃO

Até a década de 1990, as pesquisas sobre discriminação e seus impactos na saúde das populações eram escassas^1^. Desde os anos 2000, evidências sobre as relações entre discriminação, condições adversas de saúde e iniquidades relacionadas vêm se acumulando^2^. O impacto da discriminação inclui danos à saúde física e mental^3,4^, além da adoção de comportamentos de risco e menor engajamento com atividades de promoção da saúde^5^. No entanto, o conhecimento na área tem sido limitado pela insuficiência de pesquisas realizadas fora dos Estados Unidos da América^4^, ausência de análises sobre discriminação em grupos definidos por sexo, classe social, raça/etnia, entre outros^1,6^ ou ênfase em uma única forma de discriminação, principalmente a racial^7^. A literatura na área frequentemente desconsidera que determinados grupos sociais se encontram na intersecção de múltiplos eixos de opressão, os quais moldam, de maneira complexa, suas experiências e visões de mundo^8^.

A interseccionalidade é uma abordagem analítica que busca problematizar o entrecruzamento de diferentes formas de marginalização. Na área da saúde coletiva, uma abordagem interseccional destaca que as injustiças sociais não se manifestam isoladamente, mas se produzem e se reforçam mutuamente, resultando em efeitos sobre condições de saúde com significados e intensidades diversos para os variados grupos populacionais^6,9-11^. Ademais, as posições e identidades (definidas por classe social, raça, sexo etc.) moldam subjetividades, o que pode conferir aos indivíduos sentidos e percepções distintos sobre as experiências de discriminação^12^. Dessa forma, analisar a relação entre injustiças sociais e iniquidades em saúde sob uma perspectiva interseccional implica desenvolver instrumentos capazes de mensurar a discriminação em diferentes grupos interseccionais.

A construção e a avaliação de um instrumento englobam desde a formulação do arcabouço teórico subjacente até a meticulosa apreciação de suas propriedades psicométricas. Na análise psicométrica, as estruturas configural, métrica e escalar devem ser examinadas para corroborar a validade interna do instrumento. Além de analisar as diversas propriedades dessas estruturas no conjunto da amostra, cabe investigar sua invariância em diferentes grupos sociais^13,14^. É a constatação de que essas três estruturas são invariantes entre os grupos comparados que permite afirmar que as diferenças observadas entre eles são factuais, e não devidas a problemas internos aos instrumentos^14-16^. Em outras palavras, a invariância é condição *sine qua non* para se afirmar que os instrumentos de aferição estão medindo o mesmo construto, na mesma extensão e na mesma intensidade entre os variados segmentos populacionais. Quando isso não ocorre ou os itens estão sendo interpretados de maneira heterogênea, a validade e a comparabilidade de suas estimativas se tornam duvidosas^17^.

A violação de invariância se apresenta, assim, como um importante problema que pode, em parte, explicar incoerências observadas nos achados de estudos prévios. Na pesquisa de Bernardo, Bastos e Moretti-Pires^18^, por exemplo, mulheres negras de posição socioeconômica elevada apresentaram a menor média de discriminação percebida entre todos os subgrupos de estudantes universitários analisados. Em outro estudo, Lee e Turney^19^ constataram que os homens tiveram médias maiores de discriminação do que as mulheres, inclusive as hispânicas.

A Escala de Discriminação Explícita (EDE)^20^ é um instrumento que investiga a percepção de discriminação interpessoal. A EDE oferece três principais vantagens: primeiro, aborda um número substancial de situações potencialmente discriminatórias; segundo, permite que os entrevistados atribuam suas experiências com tratamento diferencial (maus-tratos) a um ou mais fatores, simultaneamente; e, terceiro, assume que os maus-tratos e sua interpretação como um evento discriminatório são construtos relacionados, mas distintos entre si.

A escala é originalmente constituída por 18 itens, em que os entrevistados que indicam ter percebido tratamento diferencial são solicitados a responder mais três subitens complementares:

1. As motivações atribuídas ao evento, tais como cor/raça, idade, classe social, entre outras;
2. O grau de desconforto relacionado com sua percepção; e
3. A interpretação do evento como discriminatório ou não.

Desde sua elaboração inicial, a EDE vem sendo submetida a diversas avaliações psicométricas^18,20-22^. Em conjunto, esses estudos sugerem que a escala tem propriedades configurais, métricas e escalares robustas. Os itens carregam consistentemente em suas respectivas dimensões com alta confiabilidade, posicionando efetivamente os respondentes ao longo do *continuum* do traço latente. As pesquisas também demonstram que a estrutura fatorial da EDE é consistente em diversas populações, desde estudantes de graduação até adultos da comunidade. Essa consistência vale tanto para sua versão autoadministrada quanto para aquela aplicada face a face.

Recentemente, Bastos et al.^23^ mostraram que duas versões abreviadas da EDE, de sete e oito itens, apresentaram boas propriedades configurais, métricas e escalares. A principal diferença entre elas reside no fato de que a versão com oito itens tem uma melhor cobertura do traço latente, mapeando melhor o *continuum* de intensidade em questão. Entretanto, não há pesquisas que avaliaram a invariância da EDE em diferentes grupos sociais*.* Assim, desconhece-se em que medida a EDE é adequada para mensurar a percepção de discriminação entre grupos e estabelecer comparações válidas entre eles. Esta pesquisa tem por objetivo avaliar a invariância fatorial das duas versões abreviadas da EDE em diferentes grupos sociais, considerando cor/raça, sexo, posição socioeconômica e suas intersecções.

## MÉTODOS

O presente estudo utilizou dados da segunda onda da pesquisa EpiFloripa Adulto, realizada em 2012. O EpiFloripa objetivou investigar determinantes sociais da saúde em uma amostra representativa de adultos (20–59 anos) da zona urbana de Florianópolis, sul do Brasil. O processo de seleção da amostra na linha de base (2009) foi realizado por conglomerados, em dois estágios, sendo a amostra final constituída por 1.720 participantes. Em 2012, todos os integrantes da linha de base foram convidados a participar de nova etapa do estudo, dos quais 1.187 foram efetivamente entrevistados.

Nas duas ondas da pesquisa, a coleta de dados se deu por meio de entrevistas face a face, conduzidas por entrevistadores previamente treinados. Para otimizar e refinar o processo, foi executado um estudo-piloto com aproximadamente 100 adultos em dois setores censitários que não fizeram parte da amostra final. O controle de qualidade foi realizado através da revisão e checagem de 15% das entrevistas, selecionadas aleatoriamente. Todas as entrevistas foram realizadas no domicílio dos participantes e as respostas, registradas com o auxílio de *Personal Digital Assistants*. Informações detalhadas sobre a coleta de podem ser encontradas em publicação anterior^24^.

### Variáveis do estudo

A amostra foi analisada segundo sexo, cor/raça, escolaridade e intersecções dessas variáveis. Utilizou-se sexo para caracterizar os participantes em homens ou mulheres. A variável cor/raça foi coletada segundo as categorias do Instituto Brasileiro de Geografia e Estatística: branca, preta, parda, amarela e indígena. Os respondentes classificados como amarelos (i.e., segmento populacional de origem asiática) e indígenas foram excluídos da análise, enquanto pardos e pretos foram agrupados numa única categoria, doravante denominada “negros”. A escolaridade foi categorizada em dois estratos de 0–11 e 12+ anos de estudo por representarem a divisão entre ensino médio e superior no Brasil. As intersecções entre essas variáveis foram avaliadas à confluência dos três grupos, sendo que as categorias decorrentes corresponderam à combinação dos respectivos estratos: intersecção de sexo e escolaridade (Homens/>11 anos de estudo; Homens/<12 anos de estudo; Mulheres/>11 anos de estudo; Mulheres/<12 anos de estudo); intersecção de cor/raça e escolaridade (Brancos/>11 anos de estudo; Brancos/<12 anos de estudo; Negros/>11 anos de estudo; Negros/ <12 anos de estudo) e intersecção de cor/raça e sexo (Brancos/Homens; Brancos/Mulheres; Negros/Homens; Negros/Mulheres).

Nossas análises têm como foco as versões abreviadas da EDE com sete e oito itens^23^. A versão de sete itens contém os seguintes: i2 (*tratado com desrespeito em lugares públicos*); i6 (*tratado como não inteligente na escola/universidade*); i7 (*tratado como pouco inteligente no estágio ou trabalho*); i9 (*avaliado injustamente em estágio ou trabalho*); i13 (*chamado por nomes que não gosta*); i14 (*excluído por amigos na escola ou universidade*); e i16 (*excluído por pessoas na vizinhança*). Por sua vez, a versão de oito itens inclui também o i15 (*excluído por colegas no estágio ou trabalho*). Todas as respostas aos itens da escala foram dicotomizadas em “sim”, quando o respondente indicou a percepção de tratamento diferencial e o atribuiu à discriminação; em todos os outros casos, os itens foram categorizados como “não”.

### Análises estatísticas

A invariância da EDE foi examinada utilizando-se duas abordagens complementares: Análises Fatoriais Confirmatórias Multigrupo (AFCMG) e o método *Alignment*. As análises baseadas nas intersecções de sexo, cor/raça e escolaridade foram conduzidas somente com o *Alignment*. De acordo com achados psicométricos prévios^20,21^, assumiu-se que os itens da EDE refletem uma estrutura configural unidimensional.

A AFCMG foi conduzida em três etapas^16,25^. Na primeira, foram estimados modelos basilares separadamente para cada grupo de interesse (i.e., homens, mulheres, brancos, negros etc.) e avaliados os índices de ajuste global RMSEA (*Root Mean Square Error of Approximation*), CFI (*Bentler’s comparative fit index*) e TLI (*Tucker-Lewis index*)^16,25^. Valores de RMSEA abaixo de 0,06 sugerem bom ajuste; valores acima de 0,10 indicam ajuste inadequado, aconselhando a rejeição do modelo. Estimativas de CFI e TLI acima ou igual a 0,95 indicam um ajuste aceitável^16^. Correlações residuais entre pares de itens foram estimadas livremente à luz dos diagnósticos interinos sugeridos pelos índices de modificação (*univariate Lagrange multiplier*), de modo a prover um melhor ajuste dos respectivos modelos basilares.

Na segunda etapa, testou-se a invariância configural, estimando-se livremente as cargas fatoriais e os limiares em cada grupo (modelo configural). A média do fator foi fixada em zero e o fator da escala, em 1, em todos os grupos. Essa última restrição foi imposta para permitir a identificação dos modelos. A terceira etapa consistiu na comparação do modelo configural com o modelo escalar, em que as cargas fatoriais e os limiares foram fixados entre os grupos. Tendo em vista que os itens da EDE eram categóricos em dois níveis, empregou-se o estimador *Weighted Least Squares Mean and Variance* (WLSMV) *adjusted* ^26^. É importante mencionar que o modelo métrico não é identificado quando os itens são binários. Isso impossibilita o teste de invariância métrica e implica que a comparação entre o modelo configural e o plenamente restrito (i.e., escalar) se dê diretamente^27^. Os modelos aninhados menos e mais restritos foram comparados pela estatística de qui-quadrado robusta, ajustada pela média e variância^28^. Um p-valor menor do que 0,05 sugere que o pressuposto da invariância entre os grupos seja rejeitado. A variação nos valores de CFI também foi tomada como indicativo de violação da invariância, quando observadas reduções acima de 0,002 na comparação de um modelo menos restritivo com um mais restritivo^26,29^.

Conforme indicado, a invariância da EDE também foi analisada segundo o método *Alignment* ^30^. Executaram-se as análises com a opção *Fixed*, recomendada ao se comparar poucos grupos. Existem duas parametrizações disponíveis com o estimador WLSMV: Theta (não padronizada) e Delta (padronizada). Visando alcançar o melhor alinhamento (de fatores a serem comparados) com um mínimo de violações (i.e., o máximo de itens plenamente invariantes), Asparouhov e Muthén^30^ propõem que se faça as análises usando ambas as parametrizações, escolhendo aquela que oferece menos *hits* de não invariância. Tendo em vista que os resultados foram intercambiáveis do ponto de vista de invariância, optou-se por apresentar somente os dados obtidos com a parametrização Theta. Os modelos foram avaliados de acordo com os mesmos índices de ajuste usados na AFCMG, descritos anteriormente. Os itens com p>0,001 na comparação das cargas e limiares entre os grupos analisados foram considerados invariantes^30^.

O processamento do banco de dados e a descrição da amostra foram realizados em Stata, versão 16.2. A AFCMG e o *Alignment* foram executados em Mplus, versão 8.8. Todas as análises estatísticas foram replicadas para as versões de sete e oito itens da EDE e levaram em conta o desenho da amostragem complexa (por conglomeração e pesos amostrais desiguais). Os *scripts* das análises estão disponíveis no material suplementar.

### Aspectos éticos

O EpiFloripa foi aprovado pelo seu respectivo comitê de ética, sob o Protocolo no 1772/11. A participação na pesquisa foi voluntária e todos os entrevistados assinaram o Termo de Consentimento Livre e Esclarecido.

## RESULTADOS

Conforme pode ser observado na Tabela 1, a amostra foi constituída por 56,9% de mulheres; cerca de 89,7% dos entrevistados se declararam brancos; e 55,3% dos entrevistados tinham até 11 anos de educação formal. Para os estratos resultantes do cruzamento de sexo, cor/raça e escolaridade, destaca-se que a maior parcela dos entrevistados (46,6%) é formada por brancos com até 11 anos de estudo. No que tange à intersecção de cor/raça e sexo e à intersecção de sexo e escolaridade, observa-se predomínio das mulheres autodeclaradas brancas (51,7%) e com até 11 anos de estudo (31,6%). Homens e mulheres autodeclarados negros (5,1% em cada grupo) foram pouco frequentes, principalmente, os de maior escolaridade (>11 anos). Esse último grupo correspondeu a apenas 1,8% do total da amostra.

**Tabela 1:** Distribuição dos respondentes conforme sexo, cor/raça e escolaridade. Florianópolis (SC), 2012.

Características	n*	%^†^	IC95%
Sexo			
Homens	504	43,1	40,5–45,8
Mulheres	683	56,9	54,2–59,5
Cor/raça			
Brancos	1061	89,7	85,9–92,7
Negros^‡^	122	10,3	7,3–14,1
Escolaridade (anos de estudo)			
>11	525	44,7	37,3–52,3
<12	659	55,3	47,7–62,7
Intersecção de sexo e escolaridade (anos de estudo)			
Homens/>11	223	19,3	16,0–23,2
Homens/<12	279	23,7	20,4–27,5
Mulheres/>11	302	25,4	21,0–30,4
Mulheres/<12	380	31,6	26,9–36,6
Intersecção de cor/raça e escolaridade (anos de estudo)			
Brancos/>11	504	43,1	35,9–50,7
Brancos/<12	554	46,6	39,7–53,6
Negros/>11	21	1,8	1,1–2,8
Negros/<12	101	8,5	5,9–12,1
Intersecção de cor/raça e sexo			
Brancos/Homens	447	38,1	35,5–40,7
Brancos/Mulheres	614	51,7	48,4–54,9
Negros/Homens	56	5,1	3,6–7,3
Negros/Mulheres	66	5,1	3,4–7,8

IC95%: intervalo de confiança de 95%.

* Frequência absoluta;

^†^ Frequência relativa, ajustada pelos pesos amostrais;

^‡^ Termo utilizado para representar o agrupamento de pretos e pardos. As variáveis cor/raça e escolaridade apresentaram, respectivamente, 4 e 3 observações ignoradas. A intersecção de sexo e escolaridade, cor/raça e escolaridade e cor/raça e sexo apresentaram, respectivamente, 3, 7 e 4 observações ausentes.

A AFCMG da versão de oito itens da EDE indicou que o modelo unifatorial teve bom ajuste aos dados em todos os subgrupos analisados, com CFI≥0,95, TLI≥0,95 e RMSEA<0,06, exceto nos participantes com 12 ou mais anos de estudo. Ao revisar os valores contidos na Tabela 2, observa-se que esse último modelo apresentou TLI limítrofe, mesmo após inclusão da correlação residual entre os itens i13 e i14. O teste de diferença do χ2 comparando os modelos configural e escalar entre participantes brancos e negros indicou que a restrição à igualdade de cargas e limiares resultou em um aumento não significativo do χ2. A variação de CFI também foi examinada, não sendo observada qualquer redução na passagem entre modelos mais e menos restritivos. Para sexo, a comparação dos modelos configural e escalar também não foi estatisticamente significante, segundo o χ2. No entanto identificou-se uma redução do CFI de 0,003. Na comparação entre participantes com até 11 e 12 ou mais anos de estudo, o teste χ2 não foi significativo, com uma variação de CFI de 0,002. Os índices de modificação não indicaram itens violando invariância em nenhum grupo analisado.

**Tabela 2:** Ajustes da Análise Fatorial Confirmatória Multigrupo para a Escala de Discriminação Explícita, de acordo com cor/raça, sexo e escolaridade. Florianópolis (SC), 2012.

	χ^2^	df	χ^2^diff	Δdf	RMSEA (IC90%)	CFit	CFI	TLI
Cor/raça								
Modelos basilares								
Brancos	47,561*	20	-	-	0,036 (0,023-0,049)	0,958	0,964	0,950
Negros	27,397	20	-	-	0,055 (0,000 - 0,102)	0,398	0,977	0,968
Invariância								
Configural	76,162*	40	-	-	0,039 (0,025-0,052)	0,909	0,965	0,950
Escalar (cargas e limiares)	81,499*	46	8,861	6	0,036 (0,023-0,049)	0,966	0,965	0,958
Sexo								
Modelos basilares								
Homens	25,771	20	-	-	0,024 (0,000-0,048)	0,966	0,989	0,984
Mulheres	31,981^†^	20	-	-	0,030 (0,005-0,048)	0,967	0,974	0,963
Invariância								
Configural	57,974^†^	40	-	-	0,028 (0,008-0,042)	0,996	0,982	0,974
Escalar (cargas e limiares)	66,785^†^	46	10,556	6	0,028 (0,010-0,041)	0,998	0,979	0,974
Escolaridade (anos de estudo)								
Modelos basilares								
>11^‡^	38,340^†^	19	-	-	0,044 (0,023-0,064)	0,661	0,959	0,940
<12	29,987	20	-	-	0,028 (0,000-0,047)	0,975	0,979	0,971
Invariância								
Configural	68,955^†^	39	-	-	0,036 (0,021-0,050)	0,953	0,969	0,955
Escalar (cargas e limiares)	72,827*	45	6,334	6	0,032 (0,018-0,046)	0,988	0,971	0,964

IC90%: Intervalo de confiança de 90%.

* p<0,001;

^†^ p<0,05;

^‡^ Modelo inclui correlação entre i14 e i13.

Nota: χ^2^ (*chi-square*); df (d*egrees of freedom*); χ^2^diff (χ^2^ *difference*); χdf (diferença entre d*egrees of freedom* do modelo configural e escalar); RMSEA (*Root Mean Square Error of Approximation*); CFit (*test of close fit* – probabilidade RMSEA = 0,05); CFI (*Comparative Fit Index*) TLI (*Tucker-Lewis Index*).

Conforme mostra a Tabela 3, os resultados obtidos com o *Alignment* foram consistentes com os da AFCMG, sugerindo invariância nos grupos definidos por sexo, cor/raça e escolaridade, separadamente. Os modelos apresentaram um bom ajuste, com CFI≥0,95, TLI≥0,95 e RMSEA<0,06, exceto no grupo com 12 ou mais anos de educação formal. Esse último modelo apresentou TLI limítrofe de 0,930. No entanto, após inclusão da correlação residual entre os itens i13 e i14, o valor de TLI foi de 0,955. O item i9 não pôde ser analisado com o *Alignment* nos grupos de cor/raça devido a problemas de estimação entre negros. Esse item apresentou cargas não padronizadas e limiares não admissíveis do ponto de vista interpretativo em função da baixa frequência de respostas positivas e do pequeno tamanho desse grupo na análise.

**Tabela 3:** Cargas fatoriais e limiares da Escala de Discriminação Explícita. Florianópolis (SC), 2012.

Item	Cargas fatoriais não padronizadas				Limiares			
	Cor/raça							
	Brancos	Negros	p-valor	R²	Brancos	Negros	p-valor	R²
i2	0,690	1,252	0,193	0,5	1,207	0,995	0,717	0,7
i6	1,026	1,694	0,387	0,5	2,042	2,417	0,704	0,7
i7	1,037	0,853	0,566	0,0	1,786	1,161	0,097	0,0
i9	0,825	8,056	0,808	0,2	1,723	10,058	0,821	0,3
i13	0,897	0,734	0,608	0,0	1,086	0,332	0,024	0,0
i14	1,067	1,026	0,899	0,9	1,493	1,531	0,915	1,0
i15	0,746	0,928	0,710	0,7	1,811	1,855	0,853	1,0
i16	0,671	0,665	0,965	1,0	2,064	1,662	0,325	0,0
	**Sexo**							
	**Homens**	**Mulheres**	**p-valor**	**R²**	**Homens**	**Mulheres**	**p-valor**	**R²**
i2	1,096	0,670	0,103	0,4	1,465	1,331	0,569	1,0
i6	1,058	1,428	0,434	0,0	2,157	2,651	0,345	0,0
i7	1,389	0,981	0,264	0,5	2,344	1,794	0,151	0,7
i9	1,077	1,099	0,865	1,0	2,095	2,109	0,887	1,0
i13	0,820	1,085	0,470	0,0	1,033	1,393	0,260	0,0
i14	1,174	1,099	0,807	0,9	1,772	1,700	0,832	1,0
i15	0,787	1,011	0,492	0,0	1,787	2,320	0,135	0,0
i16	1,062	0,618	0,140	0,4	2,437	2,133	0,382	0,8
	**Escolaridade**							
	>11* anos	**<12 anos**	**p-valor**	**R²**	**>11 anos**	**<12 anos**	**p-valor**	**R²**
i2	0,68	0,785	0,614	0,8	1,228	1,043	0,271	0,0
i6	1,025	1,135	0,741	0,8	1,965	2,091	0,723	0,9
i7	1,344	0,778	0,063	0,0	2,003	1,446	0,047	0,0
i9	0,964	0,952	0,942	1,0	1,748	1,808	0,742	1,0
i13	0,577	0,721	0,526	0,6	0,836	0,851	0,830	1,0
i14	0,817	0,797	0,901	1,0	1,156	1,377	0,262	0,7
i15	0,961	0,666	0,280	0,0	1,867	1,780	0,715	0,0
i16	0,854	0,588	0,706	0,0	2,154	1,929	0,438	0,0

Não foi possível estimar cargas e limiares para negros no item i9.

* Modelo inclui correlação entre i13 e i14.

Os dados da Tabela 4 mostram que não há qualquer violação de invariância nas intersecções de cor/raça e escolaridade, cor/raça e sexo, sexo e escolaridade com a versão reduzida da EDE de oito itens. Na intersecção entre “cor/raça e escolaridade”, o subgrupo de negros com mais de 12 anos de educação formal foi retirado das análises. A amostra substancialmente pequena (21 participantes) para esse grupo ocasionou problemas de estimação, com cargas e limiares não admissíveis do ponto de vista interpretativo e tabelas de contingências sem informação.

**Tabela 4:** Cargas fatoriais e limiares da Escala de Discriminação Explícita*,* de acordo com intersecções. Florianópolis (SC), 2012.

Item	Sexo e escolaridade									
	Cargas fatoriais não padronizadas					Limiares				
	Homens/>11	Homens/<12	Mulheres/>11^†^	Mulheres/<12	R²	Homens/>11	Homens/<12	Mulheres/>11	Mulheres/<12	R²
i2	1,251	1,154	0,544	1,003	0,5	1,826	1,099	1,088	1,116	0,0
i6	0,836	1,200	1,465	1,739	0,0	1,933	2,076	2,173	2,219	0,7
i7	1,399	1,330	1,389	0,930	0,6	2,323	2,038	1,807	1,291	0,1
i9	0,870	1,363	1,313	1,194	0,1	1,890	2,140	1,830	1,723	0,7
i13	0,759	0,759	0,776	1,205	0,0	0,956	0,876	0,923	0,939	0,8
i14	1,278	1,025	0,893	1,036	0,7	1,742	1,529	1,071	1,345	0,5
i15	0,951	0,750	1,335	1,028	0,0	1,830	1,689	2,150	1,959	0,0
i16	1,048	1,139	1,047	0,395	0,4	2,532	2,223	2,167	1,865	0,1
	**Cor/raça e escolaridade**									
	**Brancos/>11** *	**Brancos/<12**	**Negros/<12**	**R²**		**Brancos/>11**	**Brancos/<12**	**Negros/<12**	**R²**	
i2	0,704	0,800	1,569	0,5		1,247	1,084	1,137	0,8	
i6	1,020	1,118	1,512	0,8		1,951	1,989	2,370	0,7	
i7	1,281	1,001	0,561	0,0		1,926	1,621	0,933	0,0	
i9	0,900	0,830	3,236	0,4		1,681	1,660	4,530	0,3	
i13	0,583	0,933	0,594	0,0		0,856	1,013	0,297	0,0	
i14	0,840	0,922	0,936	1,0		1,147	1,409	1,465	0,8	
i15	0,961	0,741	0,670	0,0		1,848	1,815	1,613	0,0	
i16	0,865	0,664	0,643	0,3		2,215	1,965	1,868	0,0	
	**Cor/raça e sexo**									
	**Brancos**	**Brancas**	**Negros**	**Negras**	**R²**	**Brancos**	**Brancas**	**Negros**	**Negras**	**R²**
i2	1,029	0,640	1,243	1,430	0,6	1,460	1,210	1,160	1,095	0,6
i6	0,923	1,299	1.535	1,943	0,6	2,063	2,233	2,509	2,742	0,7
i7	1,310	1,044	1,287	0,769	0,5	2,370	1,657	1,618	1,158	0,0
i9	0,801	1,034	10,903	7,227	0,3	1,904	1,797	13,006	11,136	0,3
i13	0,875	1,084	0,508	0,761	0,0	1,178	1,191	0,155	0,617	0,0
i14	1,153	1,131	1,133	0,988	0,9	1,745	1,477	1,854	1,476	0,7
i15	0,712	0,993	0,935	1,150	0,6	1,766	2,064	1,643	2,708	0,3
i16	1,262	0,507	0,416	1,242	0,0	2,707	1,973	1,710	2,027	0,0

No item (i) i9, não foi possível estimar cargas e limiares para negros com até 11 anos de estudo.

* Modelo inclui correlação entre i13 e i14;

^†^ Modelo inclui correlação entre i13 e i14.

As análises descritas anteriormente foram replicadas para a versão de sete itens da EDE. Na AFCMG, os modelos basilares de cada grupo também apresentaram ajuste aceitável, com CFI≥0,95, TLI≥0,95 e RMSEA<0,06. O item i9 apresentou novamente problemas de estimação no modelo configural para os negros. O teste para comparar os modelos configural e escalar (χ2diff=11,666; Δdf=5; p=0,04) entre participantes brancos e negros mostrou um aumento significativo no qui-quadrado, sendo também observada uma redução no CFI de 0,004 entre os modelos mais e menos restritos. Para sexo e escolaridade, o teste de χ2 comparando os modelos configural e escalar não foi estatisticamente significante, mas o CFI apresentou reduções de, respectivamente, 0,004 e 0,005. Os índices de modificação foram examinados, não sendo identificados itens com violação de invariância (ver Tabela 1, material suplementar).

O método de *Alignment* também foi utilizado para avaliar a versão reduzida de sete itens da escala. Os ajustes dos modelos foram aceitáveis, com CFI≥0,95, TLI≥0,95 e RMSEA<0,06. Os valores de cargas e limiares não foram significativamente diferentes entre brancos e negros, indicando invariância entre os grupos de cor/raça. Contudo, o item i9 apresentou o mesmo problema da versão de oito itens, não sendo passível de apreciação para a comparação em questão. Para sexo e escolaridade, todos os sete itens foram considerados invariantes (ver Tabela 2, material suplementar). Os achados também mostram que os sete itens foram invariantes entre todos os subgrupos interseccionais analisados (ver Tabela 3, material suplementar), com exceção do i13 (*chamado por nomes que não gosta*), que foi considerado não invariante para a intersecção “cor/raça e escolaridade” entre o subgrupo de participantes negros com menos de 12 anos de escolaridade. O problema de estimação das cargas fatoriais não padronizadas e limiares do i9 (i.e., *avaliado injustamente em estágio ou trabalho*) se manteve na versão de sete itens.

A Figura 1 oferece uma síntese dos achados deste estudo, ilustrando que as versões abreviadas da EDE com sete e oito itens se mostraram invariantes, sendo os resultados da AFCMG e do método de *Alignment* amplamente consistentes.

**Figura 1:** Síntese dos testes de invariância da Escala de Discriminação Explícita.

*Método 1 (M1): Análise fatorial confirmatória multigrupo; ^†^Método 2 (M2): Método *Alignment*; ^‡^Estimativas referentes ao i9 não puderam ser avaliadas na análise do grupo cor/raça, incluindo sua interseção com escolaridade e sexo.

## DISCUSSÃO

As experiências discriminatórias e seus impactos sobre a saúde de variados grupos sociais vêm sendo captados por algumas escalas disponíveis na literatura^20^. A EDE, em particular, tem sido utilizada em estudos brasileiros^31,32^ e avaliações psicométricas anteriores apontam para sua validade e confiabilidade^20-22^. Ainda assim, o pressuposto fundamental de invariância de seus itens em diferentes grupos sociais e interseccionais permaneceu, até agora, sem qualquer apreciação crítica. Neste estudo, as duas versões da EDE avaliadas apresentaram invariância configural e escalar entre grupos definidos por sexo, cor/raça e escolaridade, indicando que o instrumento permite estimações de discriminação comparáveis. Nossos achados sugerem, ainda, que as duas versões abreviadas da EDE apresentam invariância em diferentes grupos interseccionais formados pelos cruzamentos de sexo, cor/raça e escolaridade. Os resultados revelaram apenas um parâmetro não invariante (limiar; item i13, *chamado por nomes que não gosta*) na versão de sete itens, indicando que a versão de oito itens é mais adequada para utilização. Isso se justifica, sobretudo, pela sua maior abrangência do traço latente, conforme evidenciado em estudo anterior^23^.

Os resultados foram consistentes ao se empregar duas abordagens metodológicas distintas (AFCMG e *Alignment*). As poucas exceções se referem ao i9 na comparação por cor/raça; intersecção “cor/raça e sexo” e “cor/raça e escolaridade”. A baixa porcentagem de respostas positivas para alguns itens já havia sido observada em publicação prévia^20^. Essa questão se mostrou ainda mais problemática na AFCMG, especialmente em um estrato esparso, como o dos negros no domínio populacional em que o estudo foi realizado. Além do problema de rarefação envolvendo o i9, para seguir com as análises de invariância, foi estimada a correlação residual entre o i14 e i13 para os grupos com mais de 11 anos de estudo; brancos com mais de 11 anos de estudo e mulheres com mais de 11 anos de estudo. Na AFCMG, também foi identificada a correlação residual entre os itens i9 e i7 para os brancos, sendo esta última observada apenas na versão de sete itens. Isso posto, nossos resultados demonstram que os dois métodos utilizados nas análises se amparam mutuamente. Contudo, o ideal seria replicar nossas análises em novo estudo, preferencialmente com tamanho amostral maior para lidar com o problema da rarefação de dados.

Em conjunto, nossos resultados dão suporte às duas versões abreviadas da EDE para investigar discriminação em diferentes grupos, definidos por sexo, cor/raça e escolaridade. Ainda assim, duas questões devem ser consideradas e enfrentadas em estudos futuros: primeiro, a proporção relativamente pequena de negros na amostra impactou as análises estatísticas; segundo, os dados analisados aqui são oriundos de adultos do município de Florianópolis, havendo, pois, a necessidade de se conduzir pesquisas adicionais para determinar se os padrões identificados emergem e se confirmam em outros domínios populacionais. O Brasil apresenta ampla diversidade cultural e sociodemográfica em toda sua extensa área territorial. Portanto, as percepções e as experiências de discriminação podem ser marcadamente diferentes em outras localidades do país, com implicações para como devem ser aferidas a partir de escalas psicométricas.

Apesar das limitações citadas e da necessidade de testes suplementares, as estratégias aqui empregadas nos permitiram concluir que as versões reduzidas da escala, propostas por Bastos et al.^23^, são invariantes entre os grupos examinados. Dessa forma, espera-se que o presente estudo contribua para a construção de uma ferramenta de qualidade que propicie a mensuração da discriminação no Brasil e no mundo em patamares mais rigorosos de validade, confiabilidade e comparabilidade.
